# Pre-sealing in different settings requires different modes

**DOI:** 10.1055/a-2740-3551

**Published:** 2025-12-03

**Authors:** Antonio Capogreco, Vincenzo Vadala', Ludovico Alfarone, Davide Massimi, Cesare Hassan, Roberta Maselli, Alessandro Repici

**Affiliations:** 1 9268 Endoscopy Unit, IRCCS Humanitas Research Hospital, Milan, Italy; 2 Department of Biomedical Sciences, Humanitas University, Milan, Italy

Intraprocedural bleeding during third-space endoscopy remains a major technical challenge, compromising visualization, increasing the risk of muscular injury, and prolonging procedural time. Although multiple electrosurgical modes are available, comparative evidence is limited, and no universal pre-sealing strategy has been established 1 .

Pre-sealing can be achieved using different electrosurgical settings in various procedural environments, such as CO _2_ or underwater. In immersion, the drop in tissue impedance reduces peak voltage and produces the so-called “frozen effect.” In contrast, in a CO _2_ environment, a similar result may be achieved using high-voltage, low-effect coagulation. However, it remains unclear whether the same pre-sealing effect can be consistently achieved across different electrosurgical settings and procedural environments.

We present the first direct comparison of forced coagulation (effect 0.3) in CO _2_ and swift coagulation (effect 3.0) in saline immersion for vessel pre-sealing, conducted during the same POEM, in different environments, with identical instrumentation and procedural setting ( Video 1 ).


Comparative vessel pre-sealing techniques during POEM using forced coagulation in CO _2_ and swift coagulation in saline, demonstrating environment-specific efficacy of electrosurgical settings in third-space endoscopy.
Video 1

Forced coagulation in CO _2_ environment produced immediate tissue whitening, consistent with the “frozen effect”, inducing effective superficial coagulation without vessel transection by minimizing spark formations ( Fig. 1 **a, c** ).

**Fig. 1:**
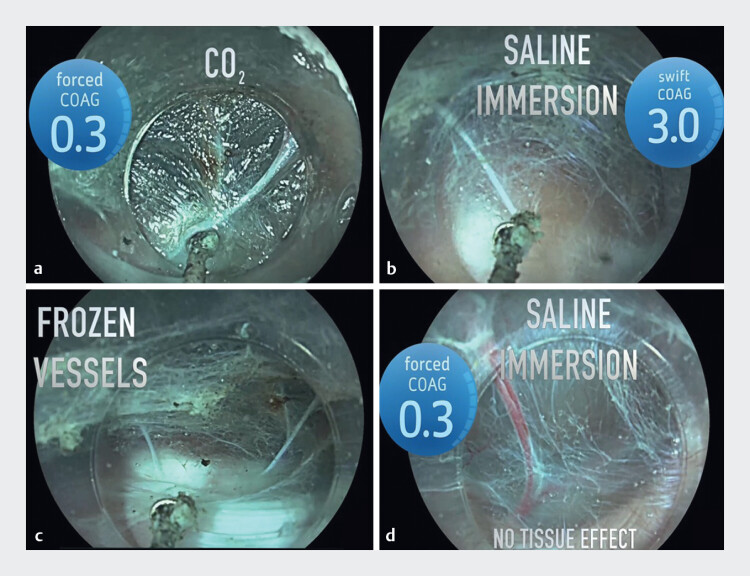
Comparative performance of forced coagulation (FC) effect 0.3 and swift coagulation (SC) effect 3.0 for vessel pre-sealing under CO _2_ and saline immersion conditions. **a** Pre-sealing achieved with FC effect 0.3 in CO _2_ . **b** Pre-sealing achieved with SC effect 3.0 in saline. **c** Frozen vessels after the application of both techniques. **d** No tissue effect after FC effect 0.3 in saline immersion.

Similarly, swift coagulation produced consistent vessel pre-sealing in saline immersion. In this low-impedance environment, the mode enables progressive tissue heating, achieving a well-defined coagulative effect with minimal cutting 2 3 ( Fig. 1 **b, c** ).

Notably, this similarity strictly depended on the appropriate environment. When transitioning from CO _2_ to saline immersion, forced coagulation at low effect failed to reproduce the frozen effect, as no coagulation occurred. This likely resulted from the drop in impedance in the conductive medium, which prevented necessary voltage buildup 4 ( Fig. 1 **d** ).

Our comparison shows that the same pre-sealing effect may be achieved with different modes in different settings. Awareness of these features supports the safety of third-space endoscopy regardless of the setting 5 . Dedicated prospective comparative studies are needed to confirm this initial observation.

Endoscopy_UCTN_Code_TTT_1AO_2AG_3AZ

## Notes

Endoscopy E-Videos https://eref.thieme.de/e-videos
*E-Videos* is an open access online section of the journal *Endoscopy* , reporting on interesting cases and new techniques in gastroenterological endoscopy. All papers include a high-quality video and are published with a Creative Commons CC-BY license. Endoscopy E-Videos qualify for HINARI discounts and waivers and eligibility is automatically checked during the submission process. We grant 100% waivers to articles whose corresponding authors are based in Group A countries and 50% waivers to those who are based in Group B countries as classified by Research4Life (see: https://www.research4life.org/access/eligibility/ ). This section has its own submission website at https://mc.manuscriptcentral.com/e-videos .
